# Claude Lévi-Strauss as a humanist forerunner of cultural macroevolution studies

**DOI:** 10.1017/ehs.2022.30

**Published:** 2022-07-12

**Authors:** Marcelo R. Sánchez-Villagra

**Affiliations:** Paleontological Institute and Museum, University of Zurich, Karl Schmid Strasse 4, 8006 Zurich, Switzerland

**Keywords:** Anthropology, ethnography, ontology, consilience, Wilson's effect

## Abstract

Cross-cultural studies of humans using methods developed in evolutionary biology and comparative linguistics are flourishing. ‘Cultural macroevolution’ has great potential to address fundamental questions of cultural transformation and human history. However, this field is poorly integrated with core cultural anthropology, although both aim in part at addressing similar issues. Claude Lévi-Strauss established a comparative approach searching for universals and documentation of diversity to bring understanding to cultural phenomena. Recognizing the nomothetic nature of Lévi-Strauss’ work, his abstraction and modelling, provides an example within anthropology of the search for universals and the study of big data, akin to cultural macroevolution studies. The latter could benefit, beyond the sophisticated analyses of big data mined from ethnographic work, from the integration with the intellectual legacy and practice of core anthropology and thus propitiate the synergistic interaction of disciplines. Attempts at rapprochement of disciplines from the natural sciences that lack pluralism and present a narrow view are deemed examples of ‘Wilson's effect’.

## Graphical abstract

**Figure:**
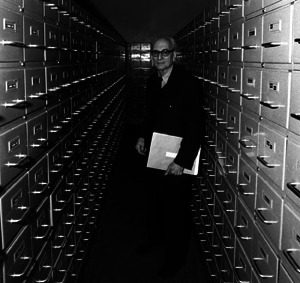

**Social media summary**: Lévi-Strauss’ anthropological work in its abstraction and modelling provides an example of the search for universals and big data analyses

## Introduction

The relatively new field of cultural evolution aims at understanding different aspects of human cumulative culture (Cavalli-Sforza & Feldman, 1981; Mesoudi, 2011; Antweiler, 2012; Lewens, 2015; Micheletti et al., 2022). The majority of studies of cultural evolution concern the processes that lead to cultural change within and among populations. Here methodological tools and concepts from psychology, cognitive biology and behavioural ecology are applied to decipher mechanisms and patterns at the level of individuals and populations, in many cases with experimental approaches. This is analogous to microevolutionary studies in biology. The approach may involve mathematical modelling, examining the gene–culture dynamics for short time spans (Mace, 2009; Creanza et al., 2017). Other studies for example concern the ‘transmission biases’ or modes of influence when information is exchanged between human groups (Kirby et al., 2008).

A less explored but significant area of cultural evolution is cross-cultural and phylogenetic studies (Youngblood & Lahti, 2018), concerning the longer-term fate of cultural phenomena or broader categorizations of cultural practices and materials over longer periods of time (Perreault, 2012; Gray & Watts, 2017; Lambert et al., 2020; Leroi et al., 2020; Lukas et al., 2021). The aim is to document the patterns and generate hypotheses explaining the processes behind the rich cultural human diversity as reflected in languages, belief systems and myths, modes of subsistence, music, kinship systems and myriad material cultural artefacts. As such, cultural macroevolution (Mesoudi, 2011) can address fundamental aspects of deep and more recent history (Smail, 2007).

In this essay I discuss how questions and approaches of cultural macroevolution studies find parallels in the work of one of the most prominent anthropologists of the twentieth century, Claude Lévi-Strauss (CLS). Recognizing these parallels raises some issues concerning anthropology and academic traditions. I refer to diverse works of CLS, including *Tristes tropiques* (1955) deemed by Susan Sontag in 1963 ‘one of the great books of our century’. Both *A view from afar*’ and *Myth and meaning* offer succinct but rich summaries in English of the main tenets of Lévi-Strauss’ prolific career.

## The search for nomothetic explanations

The study of human affairs is traditionally approached from diverse disciplines coming from both the humanities (Geisteswissenchaften) and the natural sciences (Naturwissenschaften). The following quote about anthropology is generally attributed to Alfred L. Kroeber, one of the many prominent students of Franz Boas: ‘Anthropology is the most humanistic of the sciences and the most scientific of the humanities’. Perhaps another dichotomy is more useful here. In 1894 the German philosopher Wilhelm Windelband introduced the notion of two different perspectives. Historical processes can be approached with ideographic and nomothetic explanations. Ideographic explanations are about specific events and their causes, while nomothetic ones aim at providing general principles or laws. Nomothetic explanations are ubiquitous in the natural sciences, but they also occur in the humanities. The main difference between the humanities and natural sciences may reside in the approach – whereas humanities are discursive, argue with words, natural scientists use measurement, data and quantitative analyses (Leroi, 2022). Social sciences such as economics are a third kind with elements of both (Kagan, 2007).

In his cross-cultural studies of kinship, art, forms of classification and myths, CLS compared cultural diversity and searched for nomothetic explanations, aiming at establishing ‘the intellectual unity of humankind’ (Doja, 2008: 325). He thus tried to document and account for cultural diversity while identifying commonalities and the principles that govern them. Furthermore, he introduced approaches that aimed at the study of culture becoming more mathematical and subject to abstraction and categorization.

## Structuralism and cultural change

Lévi-Strauss developed for anthropology the approach of structuralism, in which within a certain realm, parts of the structure are connected in a network in which each part makes sense and responds to all others within that system. If one wishes to understand that part by itself, one is lost, but when considering its many interactions, one can. Thus, each structure is unique and has its own network, definitions and references. Yet the principles or mechanisms that operate in structures, in the relations of parts, are universal. This universality derives from the way the ‘mind’ (neurobiology) operates, the result of biology, which follows the laws of chemistry and physics. CLS was not afraid of reductionism when leading to productive understanding.

CLS expressed his admiration for natural sciences in their mathematical and predictive power, and was instrumental in attempts to make anthropology more like them. CLS’ structuralism was inspired by linguistics, a complex and rich subject (Maniglier, 2002). Lévi-Strauss (e.g. 1969, 1978, 1985) repeatedly stated that anthropology has to become like linguistics, a more quantitative and analytical science. CLS’ structuralism was influenced by Saussure's and Jakobson's works – in them, the different parts of a language build a structure in their reciprocal relationships. From the structural phonology of Jakobson he took the concept of phonemes as inspiration to an analogous concept in kinship terms (Lévi-Strauss, 1958).

In Britain a school of ‘neo-structuralists’ developed within cultural anthropology which generally followed CLS in the comparative approach but diverged in its main goals (Kuper, 1996). The ultimate concern of CLS was to find universal principles in human culture; the functionalist British anthropologists, in contrast, were mostly trying to decipher the organization of particular societies or group of societies (Leach, 1970; Hugh-Jones, 2008) – an idiographic approach.

It has been claimed that structuralism is not transformative, it cannot explain change, as it offers no method to reconstruct the origin of a system. This is a valid critical remark – in particular to ‘British structuralism’ (Hugh-Jones, 2008). Structuralism lacks a formal, numerical method of historical reconstruction, something several kinds of phylogenetic analyses in biology provide. Yet the studies of CLS were comparative, and one of their main goals was to decipher common patterns of different systems – as in the canonical formula of myths – and with that reconstruct the ancestral one. CLS aimed at historical reconstructions and structuralism was a tool that he used pragmatically. With this approach he generated a hypothesis of historical transformation and continuity, as exemplified by the study of exchange systems in relation to kinship (Lévi-Strauss, 1969; Rosman & Rubel, 2009). As in structural linguistics examining the relationships of phonemes and not their composition (Maniglier, 2002), Lévi-Strauss’ (1969) study of kinship stressed the relationships between groups. His work developed the subjects of marriage rules and kinship terminology, now addressed with cultural macroevolution approaches (Passmore & Jordan, 2020).

The structural approach led to many different applications (e.g. Burnham, 1973), but CLS was critical of or distanced himself from many of those associated with ‘postmodernity’ (Lévi-Strauss, 1981: 641), such as Roland Barthes (Loyer, 2018). On the other hand, the general comparative approach and the general principle of the structural approach of CLS also inspired analytical works of relevance to cultural evolution studies. An example is that of how indigenous knowledge is lost with the decrease of interactions among groups owing to extinction: the impoverishment of networks of indigenous culture (Cámara-Leret et al., 2019).

## The study of myths

In his approach of structural analyses of myths, CLS identified their parts and elements in order to discover the form of relations among them and look for universal patterns of such relations. CLS used abstraction by breaking down myths into minimal units of narrative structure he called mythemes, analogous to phonemes of structural linguistics in that they exist in relation to parts of a system. Variation around mythemes concerns their becoming negated, inverted or recoded (Schwimmer, 2009). New myths are formed by putting together pieces of stories that are recycled, reused: the bricolage, leading to an almost unlimited number of combinations (Doniger, 2009). CLS performed large-scale studies of myths, making comparisons of them from quite distant geographic regions, as from different continents. He was after ‘universal cognitive biases’ (van Schaik, 2019: 87), such as those predicting responses in supernatural beliefs to large-scale problems such as social inequity, floods and droughts (van Schaik & Michel, 2016).

CLS also postulated a ‘canonical formula’ that expressed mathematically the relation of parts in myths that presumably exist universally across cultures. This formula has since then has been repeatedly examined and explored in its significance as an attempt at abstraction (Mosko, 1991; Petitot, 2016) and even at prediction (Darányi et al., 2013). Fundamentally, the canonical formula is just an instrument of expressing mathematically some simple relations of correspondence in which elements of myths can be analyzed. The challenge is to identify what those parts are and to assume relations that may be spurious – here is where the limitation of the approach lies (Turner, 2009).

As described by Philippe Descola (1996), CLS’ general approach to the study of myths is analogous to what the Achuar, an Amazonian Jivaroan group living along either side of the border between Ecuador and Peru, do with images that appear in their dreams: they reduce them to ‘minimal logical units in order to derive practical information from them’ (p. 118). The Achuar use dream elements not at face value, but to extract the logical operations that they reveal.

The peculiar associations of myths from different regions postulated by Lévi-Strauss and the lack of a transparent and reproduceable method beyond the principles of structuralism led to substantial critiques of CLS’ approach (Turner, 2009). Analytical methods have been used to study myths worldwide with a level of generality that avoids some of the issues raised by the work of CLS (Thuillard et al., 2018). The study of folktales, also following a long previous tradition of scholarship and hypotheses to test, has been productively developed using cultural macroevolutionary approaches, as in the study of oral traditions and plots associated with the story of Little Red Riding Hood (Tehrani, 2013).

An example of the power of the study of myths in cultural evolution is that concerning the history of dogs in the Americas as to their origin and past diffusion as revealed by biological data (Segura et al., 2022). The application of the neighbour-joining tree method based on Jaccard distances on a database of 23 myths concerning dogs and 22 geographic areas showed a correlation between history and geography (d'Huy, 2022). The approach hypothesized two waves of settlement in America and made it possible to reconstruct ancestral mythologies around dogs.

Lévi-Strauss aimed at understanding the ecological environment of a society and identifying the traits of the natural habitat that influence symbolic thought. CLS devoted ‘meticulous attention to the flora, fauna and astronomical and climatic cycles particular to the places from which the myths that he studies originate’ (Descola, 2009: 105). He used this information to understand sources of variation in details of myths among societies. This clarity of ideas is in contrast to the controversies about the effect of ecology on cultural phenomena, a central issue in South American anthropology (Raffles, 2002). An example of ecological determinism is the work of Betty Meggers (1971), who stated that ‘the level to which a culture can develop … is dependent upon the agricultural potentiality of the environment it occupies’ (Meggers, 1954: 815). She postulated that the cultural consequences of the environment were predictable. These ideas were tied to diffusionist ones. Any evidence of a ‘complex’ culture in the Amazonian forest could only be the result of transmission from other, richer, productive areas. Recent work has brought a deeper understanding of the importance of human agency and the complexities of the landscape and environment in which this agency operates (Hecht, 2013; Rostain & Jaimes Betancourt, 2017). This discussion should inform and enrichen important attempts within cultural macroevolution to address if ‘cultural history’ or ‘ecological environment’ determines human behaviour (Matthew & Perreault, 2016). These studies usually make use of large amounts of data and databases.

## Big data and ethnography

**Figure 1.**
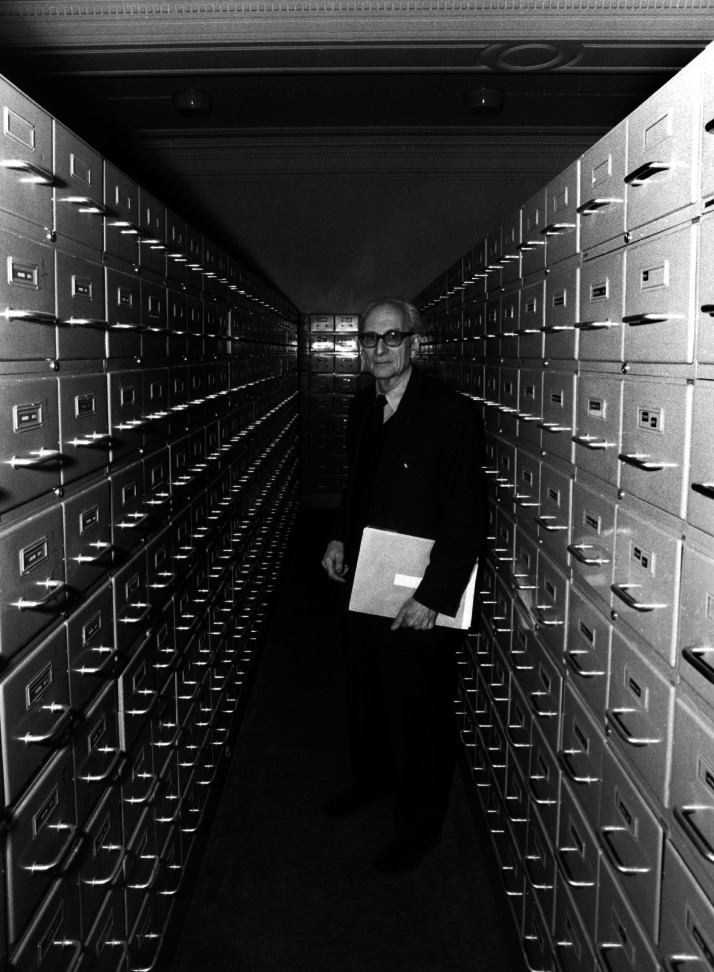
Claude Lévi-Strauss and the HRAF in Paris. Lévi-Strauss posed fundamental questions on cultural diversity and origins, and advocated extensive examination of data to address them. Keystone-France/Gamma-Keystone via Getty Images, with permission.

Cultural macroevolution studies use big data and sophisticated quantitative analyses (Evans et al., 2021). There are several global comparative cultural and linguistic databases, such as D-PLACE (https://d-place.org/), Glottobank (https://glottobank.org/) and Seshat (Turchin et al., 2015), among others. The list includes the ‘human relations area files’ (https://hraf.yale.edu/), which CLS brought to France with his special filing cabinets in the early days, long before they became digital (Figure 1). CLS became the supporter and host of the main European Center for comparative, cross-cultural ethnographic documentation (Bucher, 2010; Loyer, 2018). CLS’ work is highly relevant to understanding, within classical anthropology, research agendas that also rely on cultural diversity using big data, while building on first-hand knowledge and collaboration with ethnographers.

Gray and Watts (2017: 7846) enthusiastically advocated for cultural macroevolution studies, with the ‘application of the type of sophisticated computational methods that are often used in the biological sciences such as network analysis of reticulate evolution, epidemiological models, and phylogenetic comparative methods. These methods can be used to compare the relative importance of different factors in the distribution of traits, model the underlying dynamics of evolutionary change, and infer the history of traits.’ In fact, computational methods can be used to examine different variables in the distribution of cultural traits and model the underlying dynamics of cultural change (e.g. Ranacher et al., 2021). Graduate students of cultural evolution and related fields such as comparative linguistics are being trained to have an impressive set of quantitative skills. However, the strong focus on large analyses and methods raises issues (Grigoropoulou & Small, 2022).

In macroevolutionary studies it is as important as or more important than the methodological know-how, to gain an understanding of primary data and the fieldwork or laboratory work that produces them (Slingerland et al., 2020). A scientist cannot conduct a statistical analysis while ignoring the underlying bias of the data or how they were collected – only with that it is possible to spot if there are obvious mistakes, impossible patterns or subtle batch effects. The abstraction involved in cultural macroevolution studies presents a challenge in the integration with anthropology (Whitehouse, 2012). The categorization of cultural phenomena in traits has been identified as an important issue (Fuentes, 2006; Slingerland et al., 2020) that can be best confronted with first-hand knowledge of the primary data and their context. This approach could include a critical consideration of ethnographic data. These data could complement those just obtained from databases for cultural macroevolution studies, or as an alternative source to experimental approaches involving human response in specific situations and studies focused on specific variables (e.g. Henrich et al., 2005). The latter has been argued by Tehrani (2006: 364), stating how ‘rather than focusing on isolated subsets of cultural complexes, ethnographers attempt to situate behaviours within wider contexts of cultural meaning, event histories, and social relationships’. Another insight from long-term ethnographic studies is that they can ‘help to establish which traditions are likely to be strongly affected by social change and rates of inter-group contact, and which ones are more stable and long-lasting’ (Tehrani, 2006: 364). The dismissal of ethnographic data by most cultural macroevolution studies is at the core of the critique of Ingold (2007) of evolutionary approaches in the study of culture.

In cultural anthropology it was a rite of passage, a common entrance into the field, to spend considerable time doing fieldwork. CLS encouraged, supported and in many cases supervised many such works (Descola, 1996; Bucher, 2010). In linguistics there was an equivalent role for fieldwork work while deciphering the language of a human group, for example reconstructing the grammar of an unknown language with the help of informants. CLS spent most of his professional life conducting research that looked for nomothetic explanations of cultural phenomena, but his ethnographic work in Brazil, as described in ‘Tristes Tropiques’, provided first-hand experience of the data and their collection. In the opinion of experts, the ethnographic work of CLS itself was limited, but not in what it led to nor in the future ethnographic work it inspired (Wilcken, 2010).

## On ontologies

Whereas the Western European philosophers and historians had not transcended a particular and situated conceptual universe, the structural anthropologist approach proposed by CLS provided a much broader view on the range and nature of human experience, with ethnographic data being paramount. He was a leading intellectual figure in questioning ethnocentrism and the objectivity and universality of Western history, relativizing the ‘notion of progress and (…) the achievements of Western science and technology’ (Doja, 2006: 21). CLS’ nomothetic work was ethnological, not based on just WEIRD people – Western, educated, industrialized, rich and democratic. As such, the important work by Henrich and colleagues (e.g. Henrich et al., 2010) pointing out the biases of many evolutionary psychology works in the investigated people and the peculiarities of one cultural group is not without a long historical predecessor addressing analogous issues, among others, in CLS.

CLS was arguably the most prominent anthropologist in the second half of the twentieth century, as was Franz Boas in the first half (Zumwalt, 2019), in bringing the idea to academics and the general public in Europe and North America that ‘other cultures are not failed attempts at being you; they are unique manifestations of the human spirit’, as expressed by Wade Davis, a popularizer of ethnographic and ethnological work (www.ted.com). CLS was a great defender on academic and practical grounds of cultural diversity and of indigenous cultures. These issues mentioned above are as relevant for cultural evolution studies as they are to classical anthropology of course – it is the same people and cultures that are a stake.

CLS’ structuralism attempts to understand a society in its own terms, notwithstanding the conceptual baggage that a Western scientist brings to research – thus the importance of the work on ontologies and of perspectives (Wagner, 2012). Situating oneself in another human perspective is challenging. Perhaps the comparison with accomplishing this across animal species is appropriate. In a highly influential paper, the philosopher Tomas Nagel (1974) argued that even when knowing all the details of a bat's sensory biology, it is impossible for us humans to ‘see’ the world as a bat, not being one oneself.

The subject of ontologies greatly developed in anthropology following CLS’ work, especially in scholarship on Amazonia. This includes the revision of animism (Descola, 2013) and perspectivism (e.g. Viveiros de Castro, 2014), with variations on the conceptualization of the world, humans and other living beings revolving around the dualism body–soul, anthropomorphism and intentionality across living beings (Ingold, 2000; Fausto, 2020). It remains to be seen how cultural macroevolution studies can incorporate these fundamental explorations of human cultural diversity in its workings.

CLS argued for universals in the way humans develop systems of thoughts and symbols. At least this helps to put all cultures on equal ground – each cultural system, with its logic derived from universal principles of the human ‘mind’ (meaning cognition, as determined by neurobiology and physiology) and celebrating and documenting the individual cases in their own right – notwithstanding the true and multivariate issues involved in any ethnographic work.

The consideration of different ontologies is important to understand cultural macroevolutionary phenomena that a purely Western science approach misses, as in the consideration of domestication by Jared Diamond (1997). Diamond argued for the potential of species to become domesticated based on biological features and the geographical distribution of such species. As important as these are, studies have shown that cultural perspectives are more likely to explain the lack or low number of domestic species in some regions of the world (Descola, 1994). Some South American canids fulfill the criteria for becoming domesticated but they have not been domesticated (Segura & Sánchez-Villagra, 2021). Likewise, multidisciplinary teams have explained how a dog-centred perspective can provide an insightful view to understand human cultural transformation and health in history (Sykes et al., 2020).

CLS argued that a rational approach of fundamentally the same kind was involved in the scientific developments of Western societies after the Renaissance and is involved in the operations of Indigenous people, as it was also involved in the Neolithic transition when domestication of plants and animals was developed, surely non-trivial cultural processes of rational thinking and planning, as were also those involving in the invention of ceramics (Lévi-Strauss, 2020).

## Dual inheritance theory

Dual inheritance theory is an important and foundational aspect of ‘cultural evolution’. It postulates that genetic and cultural evolution are intertwined, they interact (Boyd & Richerson, 1980). The transmission of cultural traits is via social learning, and the mechanisms of genetic transmission are those figured out by evolutionary biologists. According the dual inheritance theory, cultural traits can be biased by genetic imperatives (Chekalin et al., 2018), and the same applies to genetic evolution as influenced by cultural traits (Mace, 2009).

Lévi-Strauss (e.g. 1985) recognized that the relation between genetic and cultural evolution is reciprocal. He stated how culturally mediated environments result in selective pressures that drive genetic evolution. He stressed the impact of culture on biological evolution (p. 14): ‘the cultural forms adopted in various places by human beings, their ways of life in the past or in the present, determine to a very great extent the rhythm of their biological evolution and its direction’. As discussed by Loyer, (2018: 525), CLS ‘thus brought the biological and the cultural together but reversed the dynamic of determination of the old physical anthropology: it was not race that dictated culture, but cultural factors that sometimes influenced the course of natural selection’. Likewise, Lévi-Strauss (1985) recognized in his controversial essay on ‘race and culture’ of his book *A view from afar* how the evolutionary history of some populations has led to biological traits more suitable to some kinds of environments than to others.

## Lost in translation and failed consilience

CLS’ concern with shared, universal workings of humans was based on empirical work, as in many of today's cognitive psychologists and neurobiologists. It is ironic that his writings ‘helped to make possible modernist ideas of deconstruction, reflexivity, and the transient nature of culture and identity’ (Doja, 2006: 18). Many, unaware of CLS’ work and ramifications, have wrongly aligned CLS with critical theory. This is a brand of anthropology that cultural evolutionists have seen as detrimental to the field or to any consilience of naturals and social sciences (van Schaik, 2019). In general, leaders of the cultural evolution field take a dismal view of social anthropology (e.g. Mesoudi et al*.,* 2006, see Tehrani, 2006 response).

CLS has had many critics within anthropology, not surprisingly given his long and prolific career. Rice (2017: 163) in his leading text on current ethnomusicology lumped CLS with ‘theorists’ and ‘postmoderns’ such as Adorno, Durkheim, ‘and more recently’ with Foucault. In *Cooking, cuisine and class*, Jack Goody (1982: 23) extensively criticized aspects of CLS work he identified with ‘Hegelian metaphysics’ (which CLS 1985 explicitly contradicted in *A view from afar*). The latter is an example of how some Anglo-Saxon anthropologists have related the Frenchman CLS to a brand of continental philosophy they deem detrimental to rational understanding, in this case wrongly. Different intellectual traditions may indeed be behind the well-documented differences between CLS and the British structuralists (Hugh-Jones, 2008). It is CLS’ insights into the different systems of thought across societies, with different inner logics, and his questioning of Western thought hegemony that paved the way for the postmodernism movement, with which he did not identify (Lévi-Strauss 1981: 641). Too simply put, CLS’ work was not ‘postmodern’, if one wishes to use such a crude and abused term.

The search for the ancestral myths (never truly managed, as discussed by Turner 2009) and other intellectual pursuits in CLS’ studies of indigenous people could be interpreted both as indicative of a naive ethnographic analogy (Currie, 2016) and as taking ancestral as ‘primitive’ in a value system of progress. Both notions are wrong. The misreading of some of the translations of his work lack the ironic twist of the use of ‘primitive’ and ‘savage’ in some of his writings (Loyer, 2018). A telling example of an attempt to correct this: the recent translation of *La pensée sauvage* (2020) by Jeffrey Mehlman and John Leavitt is entitled *Wild thought* and not *The savage mind*, as in the 1966 version.

## Historical and societal sensitivity in cultural evolutionary studies – on Darwin

Many prominent practitioners in the field of cultural evolution have not been afraid to express explicit association of the field with ‘Darwinian evolution’. The subtitle of Mesoudi's (2011) important ‘cultural evolution’ textbook is *How Darwinian theory can explain human culture and synthesize the social sciences*. Laland's (2017) book is titled *Darwin's unfinished symphony – How culture made the human mind*. In fact, these two authors, as many others, have made significant contributions to evolutionary theory in ideas and matters that go beyond Darwin and Wallace's natural selection. Laland, for example, has led efforts to propose that current evolutionary theory is significantly different from that of the Neodarwinian synthesis of the first half of the twentieth century (Laland et al., 2014). Charles Darwin postulated evolution by natural selection without any idea of later discovered inheritance mechanisms. The Neodarwinian synthesis (and much of mainstream evolutionary biology today) has come to focus almost exclusively on genetic inheritance and processes that change gene frequencies (Mayr & Provine, 1980). The new evolutionary biology identifies numerous processes by which organisms grow and develop and influence evolution (Diogo, 2017). To call cultural evolution ‘Darwinian’ is a misnomer. Cultural macroevolution is informed by a pluralistic conceptual and methodological field, of which Darwin and Wallace were fundamental early contributors – no less, no more.

The theoretical advances in cultural evolution studies concern matters that are informed by diverse ideas and fields that came after Darwin. These include among others dual inheritance theory discussed above, as well as information on macroevolution informed by studies of extinction (Zhang & Mace, 2021), phylogenetic systematics and the comparative method (Nunn, 2011), and matters of biases of the record of the past (Perreault, 2019).

To call cultural evolution ‘Darwinian’ is surely detrimental to making it an appealing field for anthropologists. The history of the use of Darwinism in the social sciences is mostly nefarious (Diogo, 2022), and that alone justifies using another term. Discussing Charles Darwin, the man himself (Fuentes, 2021) and views in his times (Braun et al., 2017), can be controversial, but this is not the matter at hand here.

Darwin looms so large that he reshaped the history of biology and made us forget other intellectual prior giants such as Goethe (search for universals, comparisons) and Cuvier (extinction) that built ultimately on Aristotle (Leroi, 2014). That Darwin is so idolized responds surely to his insights, his ‘genius’ (Wilkins, 2009) and influence, but surely also to the cultural evolutionary process of the development of the tale of the science in which Anglo-Saxon science became dominant. To deny the brilliant and diverse insights of Darwin would be absurd, but one can easily dismiss his significantly wrong ideas on inheritance (Darwin, 1868), for example. To limit the historical references of cultural evolution to Darwin and ignore a much richer historical background is a missed chance.

For cultural evolution to contribute to an intellectual breakthrough, this field needs to synthesize and bring consilience (Shore, 2012); it should also become more sophisticated and sensitive to the issues of the past and the present. There are important historical figures within anthropology that conducted comparative work and historical research, even borrowing concepts from evolutionary biology, as in Margaret Mead's discussion on micro- and macroevolution in biology and in culture (Schwartz & Mead, 1961). Here I have argued that CLS is one such central figure in anthropology.

This essay does not aim at making of Lévi-Strauss a hero to replace Darwin. CLS argued that the glorification of individual creativity is an illusion, in different contexts, including his own persona and in his consideration of cultural transformation in general (Levi-Strauss, 1955). This included the study of Amerindian art, devoid of the individualistic self-display of Western art (Fausto, 2020), so prevalent in Western thought.

## Quo Vadis ‘cultural macroevolution’? Avoiding ‘Wilson's effect’

‘Cultural evolution’ is developing as its own field, with its own society, conference and journals. This indicates a maturity and critical mass, but it comes at a cost of a lack of integration with anthropology, when in fact it is concerned with issues that have been addressed by this discipline. The practice of cultural macroevolutionary studies could benefit from more than a century of scholarship by incorporating some aspects of the humanistic tradition of anthropology. This may not translate one-to-one in any specific method of analysis being incorporated nor in any new big-data source, but it could bring a sense of depth and scholarship and with that a more critical, nuanced and integrative practice in the discipline. It would help to bring about the consilience of natural and social sciences that has eluded previous attempts. It would avoid what I would call ‘the Wilson effect’, after the author of *Sociobiology* (Wilson, 1975) and *Consilience* (Wilson, 1998), namely what happens when there is an attempt for rapprochement coming from the natural sciences with ideas that are actually narrow in the lack of pluralism and devoid of historical nuance and consideration, leading actually to distancing among disciplines (Shweder, 2012).

Many cultural evolutionists complain of ‘postmodernity’ in anthropology – a crude and simplistic umbrella term that is actually referring to different currents and approaches in the social sciences and humanities. The integration of disciplines will require a long-run and collective effort. Integrating the cultural evolution meeting as a part or as a preceding conference to a cultural anthropology one would be a way to facilitate bringing people together.

Gray and Watts (2017: 7846) stated that ‘The combination of big(ish) data and computational methods has the potential to transform the social sciences and humanities by enabling powerful quantitative tests of hypotheses that would have previously only been analyzable in much more limited ways’. Although I concur with the enthusiasm on the importance of this approach, I think it is quite clear that ‘transformation’ (perhaps expansion is a better term) will not occur unless there is an engagement with matters and concepts developed in the social sciences and the humanities. The rich intellectual history of anthropology provides links and clues to develop such communication, and CLS is one if not the most appropriate central figure to which to refer to in this endeavour.

One may argue that the CLS abstractions and big data approach did not use evolutionary approaches. CLS did not practise ‘cultural macroevolution’ as it is understood today (Mesoudi, 2011), in the same way that Aristotle was not a scientist and Goethe was not Darwinian – it is anachronistic to use those terms in such contexts.

I much doubt that there can be a simple, straightforward integration in cultural macroevolution studies of the structuralist approach to study myths from CLS, including the identification of mythemes as units of comparison, with the standard cultural macroevolution methods. Yet CLS brought a contextual understanding of myths and a consideration of different ontologies that, although relevant, the typical cultural macroevolution approach lacks.

The main point of this essay is to emphasize the comparative and historical approaches of CLS and of many other cultural anthropologists, as ‘they force the investigators to define terms, use consistent categories, and in general discipline their data’ (Bridgeman, 2006: 351). There is a compelling case to be made that ‘It is not evolutionary models, but models in general that social science needs’ (Bridgeman, 2006). As stated by Bridgeman (2006: 351), ‘the value of the models may stem not so much from their link to evolutionary theory as from the way that they force the investigators to define terms, use consistent categories, and in general discipline their data’. It would seem that the more we learn about biology, the more multi-modelled it is; and the more we learn about culture, the more we realize that we need creative new ways to understand the data beyond standard biological models (Matsumae et al., 2021).

If a method is just a tool and not an end in itself, it follows that cultural macroevolution studies are just anthropology with new tools. Ignoring the rich historical background of studies of culture would make ‘cultural evolution’ parochial. Incorporating efforts into anthropology on the other hand would help circumvent the false association of cultural evolution with social Darwinism.

## Research transparency and reproducibility

Data availability statement: there are no data accompanying the manuscript.
